# Local adaptation of the trematode *Fasciola hepatica* to the snail *Galba truncatula*

**DOI:** 10.1051/parasite/2012193271

**Published:** 2012-08-15

**Authors:** G. Dreyfuss, P. Vignoles, D. Rondelaud

**Affiliations:** 1 INSERM U1094, Faculties of Medicine and Pharmacy 87025 Limoges Cedex France

**Keywords:** *Fasciola hepatica*, *Galba truncatula*, cercaria, experimental infection, redia, river bank, *Fasciola hepatica*, *Galba truncatula*, berge de rivière, cercaire, infestation expérimentale, rédie

## Abstract

Experimental infections of six riverbank populations of *Galba truncatula* with *Fasciola hepatica* were carried out to determine if the poor susceptibility of these populations to this digenean might be due to the scarcity or the absence of natural encounters between these snails and the parasite. The first three populations originated from banks frequented by cattle in the past (riverbank group) whereas the three others were living on islet banks without any known contact with local ruminants (islet group). After their exposure, all snails were placed in their natural habitats from the end of October up to their collection at the beginning of April. Compared to the riverbank group, snails, which died without cercarial shedding clearly predominated in the islet group, while the other infected snails were few in number. Most of these last snails released their cercariae during a single shedding wave. In islet snails dissected after their death, the redial and cercarial burdens were significantly lower than those noted in riverbank *G. truncatula*. Snails living on these islet banks are thus able to sustain larval development of *F. hepatica*. The modifications noted in the characteristics of snail infection suggest the existence of an incomplete adaptation between these *G. truncatula* and the parasite, probably due to the absence of natural contact between host and parasite.

## Abstract (fr)

Six populations de *Galba truncatula*, vivant sur des berges de rivière, ont été soumises à des infestations expérimentales avec *Fasciola hepatica* pour déterminer si leur faible sensibilité au Digène est due à la rareté ou à l’absence de contacts naturels entre ces mollusques et le parasite. Les trois premières populations proviennent de rives fréquentées par les bovins dans le passé (groupe berges de rivière), tandis que les trois autres vivent sur des berges d’îlots pour lesquelles aucun contact connu avec les ruminants locaux n’a été rapporté (groupe îlots). Après leur exposition, tous les mollusques ont été remis dans leurs habitats naturels à la fin du mois d’octobre jusqu’à leur récolte au début d’avril. Par rapport au groupe berges de rivière, les mollusques infestés qui meurent sans émission cercarienne prédominent nettement dans le groupe îlots, tandis que les limnées avec émission sont peu nombreuses. La plupart de ces dernières émettent leurs cercaires en une seule vague. Lors de la dissection des cadavres du groupe îlots, les charges rédienne et cercarienne sont significativement plus faibles que celles notées dans l’autre groupe. Les limnées vivant sur les berges de ces îlots sont donc capables d’assurer le développement larvaire de *F. hepatica*. Les modifications que l’on note dans les caractéristiques de l’infestation suggèrent l’existence d’une adaptation incomplète entre ces *G. truncatula* et le parasite, probablement en raison de l’absence de contacts naturels entre l’hôte et le parasite.

According to the Red Queen hypothesis, parasites should be better at infecting sympatric (local) populations of snails than allopatric host populations (Greischer *et al.*, 2007; King *et al.*, 2011). Among the diverse investigations reported in the literature, parasite have been found to be more infective to their sympatric hosts in several systems (Lively *et al.*, 2004; Muñoz-Antoli *et al.*, 2010), thus demonstrating local adaptation between both partners. This finding was also noted in the model *Galba truncatula*- *Fasciola hepatica*. In the French region of Limousin, the highest rates of experimental infections (> 50 %) with *F. hepatica* were noted for snail populations living in open drainage networks of swampy meadows and in road ditches which border them (Rondelaud, 1993). According to this author, this result suggests local adaptation of snails to parasite and can be explained by the great frequency of natural encounters between *G. truncatula* and the parasite in these sites so that these snail populations are locally adapted to *F. hepatica*.

Contrary to the meadow populations of *G. truncatula*, experimental infections of snails living along river and brook banks resulted in a high mortality (> 70 %) at day 30 post-exposure and a low prevalence of infection (< 35 %) with *F. hepatica* (Rondelaud, 1993; Rondelaud & Dreyfuss, 1996). Besides, the number of *F. hepatica* cercariae produced by these *G. truncatula* was low: a mean of 32.1 to 93.2 per snail according to snail population used for experimental infections (Rondelaud, 1993; Vignoles *et al.*, 2011). To explain this poor susceptibility of snails to parasites, the first explanation proposed was to relate this finding to drastic characteristics of sites in which these *G. truncatula* were living. Indeed, these habitats were few in number (2.1 % out of 7,709 sites found in the region of Limousin: Vareille-Morel *et al.*, 2007) and scattered along the banks of the different rivers, which crossed this region (Dreyfuss *et al.*, 1997). Their area was often reduced (< 5 m2) and snail density was low (< 10 overwintering *G. truncatula* per habitat in March-April) with a single annual generation (Rondelaud *et al.*, 2009, 2011). However, the poor conditions of snail life in these riverbank habitats were not probably the single factor to explain the maladaptation of these snails to local *F. hepatica* and one may wonder if the scarcity or the absence of natural contacts between these *G. truncatula* and the presence of naturallyinfected definitive hosts in their habitats would not have an influence on this parameter.

This hypothesis was supported by the fact that domestic ruminants in central France did not have access to brooks, ponds and rivers since 1970 for their drinking so that their watering was often made by the use of fixed or mobile tanks (Rondelaud *et al.*, 2009). To answer this question, snails originating from three banks frequented by cattle in the past for their drinking (riverbank group) were subjected to experimental infections with *F. hepatica*. The results were compared to those noted in three other snail populations living along the banks of two islets (no known contact of snails with wild and domestic ruminants) and infected according to the same protocol (islet group). To avoid eventual modifications in larval development of the parasite with snail breeding in the laboratory, the *G. truncatula* were raised in their natural habitats from October 2009 until the beginning of April 2010.

Table I indicates, for each snail habitat, its eventual relationships with cattle in the past, its geographical coordinates, and the number of preadult snails (3-3.5 mm in height) collected at mid-October. These six snail habitats were located along the Creuse River on the communes of Ruffec, Saint-Gaultier and Thenay, department of Indre (central France). The maximum distance between the sites 1 and 6 was 22 km. No natural infection with *F. hepatica* or with another digenean was noted in the six populations from 1980 until 2008, in spite of annual or biennial collections and dissections of 10-20 adult snails per site (according to snail density) performed at the end of May. The soil of these habitats was devoid of macrophytes and was composed of silt and sand with rocks (site 1) or shingle (the other sites), supported by calcareous subsoil. The pH of running water ranged from 6.9 to 7.6 throughout the year with 28-34 mg/L of dissolved calcium. Three sites (1-3) were frequented by cattle in the past for water drinking, while the three others (4-6) have had no contact known with local ruminants because of their location around an islet. The presence of coypu was not still reported in the part of the river concerned by the location of these six sites and no print of other small mammals was found during investigations made by our team since 1980. These six habitats were submersed by running water from mid-October 2009 until the end of March 2010. Eggs of *F. hepatica* were collected in the gall bladders of local cattle (limousine breed) at the slaughterhouse of Limoges (France). They were washed several times with spring water and were incubated for 12 days at 24 °C in the dark (Ollerenshaw, 1971).

**Table I.** Main characteristics of the six snail habitats studied along the Creuse River in the department of Indre (central France).

Type of snail habitats	Place and French commune (site no.)	GPS coordinates	Date of the last frequentation of the site by cattle	Number of snails collected
River banks frequented by cattle in the past (riverbank group)	La Renauderie, Thenay (site 1)	46°37’37”N, 1°27’34”E	1969	137
	La Ribère, Thenay (site 2)	46°38’30”N, 1°26’54”E	1982	101
	Le Gué du Moulin, Thenay (site 3)	46°38’90”N, 1°25’56”E	1977	88

Islet banks without contact known with ruminants since 1900 (islet group)	Left bank of “l’Ilon”, Saint-Gaultier (site 4)	46°37’53”N, 1°25’20”E	–	194
	Right bank of “l’Ilon”, Saint-Gaultier (site 5)	46°37’59”N, 1°25’17”E	–	92
	Left bank of an islet, Ruffec (site 6)	46°37’36”N, 1°10’22”E	–	117

The whole snails present in each habitat were collected at the beginning of October (their shell height was 3-3.5 mm at that time) by two persons during 20-30 minutes and the site was verified two times to detect any snail which has escaped to the first investigation. They were then subjected to individual bimiracidial exposures with *F. hepatica* at 18 °C for four hours. After exposure, snails were directly replaced in their respective habitats (at the rate of 10 per 1-m length of bank) without snail marking or snail placing into cages. At the beginning of April 2010, the *G. truncatula* were collected from each site and were individually placed in 35-mm Petri dishes with pieces of dead grass, lettuce and spring moss according to the method by Rondelaud *et al.* (2007). These dishes were progressively acclimatized at a constant temperature of 18 °C for three days and were later placed in an air-conditioned room under the same temperature and natural photoperiod. This temperature was selected for limiting snail mortality in these groups, which were subjected winter conditions. A daily surveillance was performed to change water and food, if necessary. If metacercariae were present, they were counted and removed from the Petri dish. This surveillance was applied up to the death of each snail. A dissection of each cadaver was then performed under a stereomicroscope to count free rediae, intraredial cercariae and free cercariae within the snail body.

The first two parameters were snail survival just after their recapture at the beginning of April and prevalence of *F. hepatica* infection (calculated in relation to the number of surviving snails). Prevalence was calculated by adding the number of cercariae-shedding snails (CS snails) and that of infected individuals which died without exit of cercariae (NCS snails), and by dividing this number by the number of recaptured snails. For each parameter, the differences were analyzed using a χ^2^ test. The other parameters for infected snails were their shell height at death, the length of the patent period, the total number of metacercariae, and that of shedding waves. The total burden of free rediae, the number of cercariae-containing rediae, the quantity of intraredial cercariae, and that of free cercariae noted in dissected snails were also taken into account. Individual values recorded for these last eight parameters were averaged and their standard deviations were established for each snail group. One-way analysis of variance and Kruskal-Wallis test were used to establish levels of significance. As most differences between the values noted for each parameter in the three populations of each group were not significant, statistical analyses were only performed for differences between the values noted for snails living at Thenay (riverbank group) and those coming from Ruffec and Saint Gaultier (islet group). All the statistics were made using the Statview 5.0 software.

Survival of *G. truncatula* just after their recapture in April (Table II) ranged in the same scale of percentages and no significant difference between these rates was noted. A similar finding was also noted for the prevalence of *F. hepatica* infection in snails. However, the number of CS snails was greater in the riverbank group, whereas that of NCS snails clearly predominated in the islet snails. The difference between the shell heights of infected snails at their death was insignificant. A similar finding was also noted for the length of the patent period. In contrast, the numbers of larvae were significantly greater in the riverbank group, whatever redial and cercarial categories. If the different types of cercariae produced by the snail are added, total cercarial production was 3.3 times greater in the riverbank group than in the other *G. truncatula*.

**Table II.** Main parameters of *F. hepatica* infection in riverbank *G. truncatula* after their collection in April 2010.

	Snail groups from		
Parameter	Thenay (riverbank group)	Ruffec/Saint-Gaultier (islet group)	Statistics
Number of snails at exposure	326	403	–
Number of surviving snails just after their recapture in April (%)	101 (30.9)	115 (28.5)	NS
Number of CS snails	29	11	–
Number of NCS snails	15	40	–
Prevalence of *F. hepatica* infection (%)	43.5	44.3	NS
Mean height (SD) of infected snails at their death (mm)	5.9 (0.7)	5.7 (0.9)	NS
Mean length (SD) of the patent period (days)	14.3 (7.1)	10.2 (6.2)	NS
Mean numbers (SD):						
• Free rediae	21.4 (5.7)	12.8 (5.6)	F = 53.69, P < 0.001
• Free rediae containing cercariae	13.8 (3.6)	5.9 (3.4)	F = 119.84, P < 0.001
• Intraredial cercariae	31.2 (13.2)	12.1 (4.5)	F = 3.93, P < 0.05
• Free cercariae within the snail’s body	46.5 (28.9)	9.9 (9.8)	F = 71.98, P < 0.001
• Metacercariae	71.0 (38.5)	22.8 (15.6)	F = 15.99, P < 0.01
Mean total cercarial production	148.7	44.8	–

CS snails, cercariae-shedding snails; NCS snails, snails infected but without cercarial shedding; NS, not significant.

Table III gives the number of shedding waves recorded during the patent period of CS snails. Most snails from the islet group shed their cercariae during a single wave and died after. In contrast, cercariae shed by most snails of the riverbank group were released during 2, 3, or 4 waves.

**Table III.** Number of shedding waves noted during the patent period in the snail groups from Ruffec/Saint-Gaultier and Thenay.

Number of shedding waves	Thenay (n = 29)	Ruffec/Saint-Gaultier (n = 11)
1	3	10
2	9	1
3	12	0
4	4	0
5	1	0

n, total number of cercariae-shedding snails.

As survival of overwintering *G. truncatula* at the beginning of April (Table II) was 30.9 % in the riverbank group and 28.5 % in islet snails, there was a low recapture rate among experienced snails during winter months. This finding disagrees with values reported by several authors on snail densities in their natural habitats during winter because the numbers of *G. truncatula* remained low and stable enough from November to March, either in swampy meadows and road ditches on acid soil (Rondelaud, 1977; Rondelaud & Mage, 1992) or along river banks upstream from a dam (Hourdin *et al.*, 2006). As no study was carried out on the survival of snails during winter months in the Creuse River in 2009-2010, it is difficult to specify if this low recapture rate is due to environmental conditions, *F. hepatica* infection, or both.

Compared to snails originating from sites frequented by cattle in the past, the *G. truncatula* coming from islet banks are also able to sustain larval development of the parasite. However, these islet snails showed several modifications in the characteristics of *F. hepatica* infection: i) the high number of NCS snails which died without cercarial shedding; ii) the redial burden and cercarial production were significantly lower than those noted in the riverbank group; and iii) a single shedding wave for most CS snails. These three findings suggest the existence of an incomplete adaptation between islet snails and the parasite, as demonstrated by Rondelaud (1993), Vignoles *et al.* (2002) for other riverbank populations of *G. truncatula* experimentally infected with *F. hepatica*.

*A contrario*, the above results confirmed the necessity of frequent natural contacts between the population of snails and *F. hepatica* for having a normal development of the parasite within the snail (Rondelaud & Dreyfuss, 1996), thus strongly supporting the coadaptation of local hosts and parasite. However, this interpretation disagreed with other data noted by our team because Spanish isolates of *F. hepatica* miracidia, coming from cattle infections, were more infective to French populations of *G. truncatula* than miracidial isolates originating from central France (Gasnier *et al.*, 2000). A higher prevalence of *F. hepatica* infection and a greater cercarial production were also noted in allopatric combinations of French snails and Moroccan miracidia (Goumghar *et al.*, 2001). In our opinion, the findings reported by the above authors might be interpreted as the consequence of a strategy developed by the parasite when it infects snail populations other than its usual intermediate hosts.

In conclusion, if the populations of *G. truncatula* used in the present study have had no natural contact with infected herbivores since the three last decades (riverbank group) or at least 1900 (islet snails), they are able of sustaining larval development of the parasite, even if several characteristics of snail infection have changed.

## References

[R1] Dreyfuss G., Vareille-Morel C. & Rondelaud D. Les habitats de *Lymnaea truncatula* Müller (Mollusque) le long de deux rivières. *Annales de Limnologie*. International Journal of Limnology, 1997, 33, 67–72.

[R2] Gasnier N., Rondelaud D., Abrous M., Boulard C., Carreras F., Diez-Banos P. & Cabaret J. Allopatric combination of *Fasciola hepatica* and *Lymnaea truncatula* is more efficient than sympatric ones. International Journal for Parasitology, 2000, 30, 573–578. 10779569 10.1016/s0020-7519(00)00038-2

[R3] Goumghar M.D., Dreyfuss G., Rondelaud D., Benlemlih M. & Cabaret J. More efficient allopatric combinations of *Fasciola hepatica* and *Lymnaea truncatula* due to modification of redial development? Parasitology Research, 2001, 87, 1016–1019. 11763431 10.1007/s004360100493

[R4] Greischar M.A. & Koskella B. A synthesis of experimental work on parasite local adaptation. Ecology Letters, 2007, 10, 418–434. 17498141 10.1111/j.1461-0248.2007.01028.x

[R5] Hourdin P., Vignoles P., Dreyfuss G. & Rondelaud D. *Galba truncatula* (Gastropoda, Lymnaeidae): effects of daily water-level variations on the ecology and ethology of populations living upstream of a dam. *Annales de Limnologie*. International Journal of Limnology, 2006, 42, 173–180.

[R6] King K.C., Jokela J. & Lively C.M. Trematode parasites infect or die in snail hosts. Biology Letters, 2011, 23, 265–268. 20961880 10.1098/rsbl.2010.0857 PMC3061183

[R7] Lively C.M., Dybdahl M.F., Jokela J., Osnas E.E. & Delph L.F. Host sex and local adaptation by parasites in a snailtrematode interaction. American Naturalist, 2004, 164, S6–S18. 10.1086/424605 15540142

[R8] Muñoz-Antoli C., Marin A., Trelis M., Toledo R. & Esteban J.C. Sympatric and allopatric infections of the planorbid snail *Gyraulus chinensis* with miracidia of *Euparyphium albuferensis* (Trematoda: Echinostomatidae). Journal of Helminthology, 2010, 84, 420–424. 20236558 10.1017/S0022149X10000143

[R9] Ollerenshaw C.B. Some observations on the epidemiology of fascioliasis in relation to the timing of molluscicide applications in the control of the disease. The Veterinary Record, 1971, 88, 152–164. 5102169 10.1136/vr.88.6.152

[R10] Rondelaud D. L’évolution démographique de *Lymnaea (Galba) truncatula* Müller en Haute-Vienne. À propos de quatre années d’observations. Annales de Parasitologie Humaine et Comparée, 1977, 52, 511–520. 603201

[R11] Rondelaud D. Variabilité interpopulationelle de l’infestation fasciolienne chez le mollusque *Lymnaea truncatula* Müller. Influence du contact préalable de la population avec le parasite. Bulletin de la Société Zoologique de France, 1993, 118, 185–193.

[R12] Rondelaud D. & Dreyfuss G. Variabilité de l’infestation fasciolienne chez *Lymnaea truncatula* Müller par rapport à la localisation de ses gîtes sur les réseaux hydrographiques. Bulletin de la Société Française de Parasitologie, 1996, 14, 189–194.

[R13] Rondelaud D. & Mage C. *Lymnaea truncatula* Müller : les conséquences d’une seule génération annuelle sur les caractéristiques de l’infestation par *Fasciola hepatica* L. Revue de Médecine Vétérinaire (Toulouse), 1992, 143, 843–846.

[R14] Rondelaud D., Vignoles P. & Dreyfuss G. La Limnée tronquée, un mollusque d’intérêt médical et vétérinaire. PULIM, Limoges, 2009, 209 p.

[R15] Rondelaud D., Fousi M., Vignoles P., Moncef M. & Dreyfuss G. Optimization of metacercarial production for three digenean species by the use of Petri dishes for raising lettuce-fed *Galba truncatula*. Parasitology Research, 2007, 100, 861–865. 17061111 10.1007/s00436-006-0353-2

[R16] Rondelaud D., Hourdin P., Vignoles P., Dreyfuss G. & Cabaret J. The detection of snail host habitats in liver fluke infected farms by use of plant indicators. Veterinary Parasitology, 2011, 181, 166–173. 21524858 10.1016/j.vetpar.2011.03.056

[R17] Vareille-Morel C., Dreyfuss G. & Rondelaud D. Les habitats des Lymnaeidae sur sol acide. À propos de quelques observations dans la région Limousin sur une trentaine d’années. MalaCo, 2007, 4, 143–147.

[R18] Vignoles P., Dreyfuss G. & Rondelaud D. Larval development of *Fasciola hepatica* in experimental infections: variations with populations of *Lymnaea truncatula*. Journal of Helminthology, 2002, 76, 179–183. 12015832 10.1079/JOH2002112

[R19] Vignoles P., Rondelaud D. & Dreyfuss G. Characteristics of *Fasciola hepatica* infections in *Galba truncatula* originating from riverbank populations. Journal of Helminthology, 2011, 85, 28–32. 20388248 10.1017/S0022149X10000246

